# Proton linear energy transfer and variable relative biological effectiveness for adolescent patients with Hodgkin lymphoma

**DOI:** 10.1259/bjro.20230012

**Published:** 2023-02-15

**Authors:** Laura Ann Rechner, Maja V Maraldo, Edward AK Smith, Anni Y. Lundgaard, Lisa L. Hjalgrim, Ranald I. MacKay, Adam H. Aitkenhead, Marianne C. Aznar

**Affiliations:** 1 Department of Oncology, Rigshospitalet, Copenhagen, DK; 2 Niels Bohr Institute, University of Copenhagen, Copenhagen, DK; 3 Division of Cancer Sciences, School of Medical Sciences, Faculty of Biology, Medicine and Health, The University of Manchester, Manchester, UK; 4 Christie Medical Physics and Engineering, The Christie NHS Foundation Trust, Manchester, UK; 5 Department of Pediatric Hematology and Oncology, Rigshospitalet, Copenhagen, DK; 6 Clinical Trial Service Unit, Nuffield Department of Population Health, University of Oxford, England, United Kingdom

## Abstract

**Objectives:**

Proton therapy has a theoretical dosimetric advantage due to the Bragg peak, but the linear energy transfer (LET), and therefore the relative biological effectiveness (RBE), increase at the end of range. For patients with Hodgkin lymphoma, the distal edge of beam is often located within or close to the heart, where elevated RBE would be of potential concern. The purpose of this study was to investigate the impact of RBE and the choice of beam arrangement for adolescent patients with mediastinal Hodgkin lymphoma.

**Methods:**

For three previously treated adolescent patients, proton plans with 1–3 fields were created to a prescribed dose of 19.8 Gy (RBE) in 11 fractions (Varian Eclipse v13.7), assuming an RBE of 1.1. Plans were recalculated using Monte-Carlo (Geant4 v10.3.3/Gate v8.1) to calculate dose-averaged LET. Variable RBE-weighted dose was calculated using the McNamara model, assuming an α/β ratio of 2 Gy for organs-at-risk.

**Results:**

Although the LET decreased as the number of fields increased, the difference in RBE-weighted dose (Δdose) to organs-at-risk did not consistently decrease. Δdose values varied by patient and organ and were mostly of the order of 0–3 Gy (RBE), with a worst-case of 4.75 Gy (RBE) in near-maximum dose to the left atrium for one plan.

**Conclusions:**

RBE-weighted doses to organs-at-risk are sensitive to the choice of RBE model, which is of particular concern for the heart.

**Advances in knowledge:**

There is a need to remain cautious when evaluating proton plans for Hodgkin lymphoma, especially when near-maximum doses to organs-at-risk are considered.

## Introduction

Early-stage Hodgkin lymphoma has an excellent prognosis with combined therapy.^1^ Radiotherapy is consolidative, and modern radiotherapy only treats the initially involved nodes.^2^ However, in recent years, there has been a trend toward using less radiotherapy,^2^ especially for young patients, due to the risk of late toxicity, such as heart disease and second primary cancers.^3–6^ One approach to reducing the radiation dose to organs at risk (OARs) for patients with mediastinal lymphoma is deep-inspiration breath hold, which has been thoroughly investigated and widely implemented for the adult population^2,7^ and is under investigation for the pediatric population.^8^

Another technique that has the potential to benefit this patient group and reduce the risk of late toxicity is proton therapy. Proton therapy is an attractive modality for young patients due to the dosimetric advantages of the Bragg peak,^9,10^ which often enables a reduction in the dose to normal tissue and therefore the risk of side-effects.^11,12^ Current practice in proton therapy is to use a constant relative biological effectiveness (RBE) factor of 1.1,^13,14^ which was chosen based on radiobiological experiments.^13,15^ However, it is known that a single value of 1.1 is a simplification, and that RBE is dependent on factors such as dose, tissue (α/β), endpoint, and linear energy transfer (LET).^16–19^ There are a few studies suggesting clinical evidence of a variable RBE (vRBE) effect for proton therapy,^20,21^ and there is a growing effort to model vRBE for proton therapy^22–27^ and investigate its potential impact.^28–35^ While proton therapy appears to have dosimetric advantages for this patient group,^36–38^ it is unknown how much vRBE impacts the dose for adolescent patients with mediastinal Hodgkin lymphoma.^36^

While the biological models for vRBE are quite uncertain, LET is a physical parameter of the protons and can be scored during Monte–Carlo simulations. An increase in LET corresponds to an increase in RBE,^14^ so some have proposed reviewing LET distributions and avoiding high LET in OARs to reduce the risk of unintentional overdosage.^39^ In current clinical practice, this can be achieved by manually replanning with different beam angles or with beam-specific planning volumes to avoid protons stopping in the region of concern.^40–42^ Moreover, incorporation of LET as an optimization parameter has been investigated.^43–46^

In this study, we investigated the LET and vRBE-weighted dose distributions for three adolescent patients with mediastinal Hodgkin lymphoma for different beam arrangements with a focus on dose to organs at risk (OARs). We hypothesized that increasing the number of fields would decrease the near-maximum LET and consequently decrease the near-maximum dose to OARs.

## Method and materials

### Patient data

For this retrospective analysis, we selected three previously treated adolescent pediatric patients with mediastinal Hodgkin lymphoma. Patients were treated with photons as part of the TEDDI protocol (radio**t**h**e**rapy **d**elivery in **d**eep **i**nspiration for pediatric patients − a NOPHO feasibility study, Danish Ethical Committee H-16035870, clinicaltrials.gov NCT03315546).^8^ The patients were the first three consecutive patients treated with radiotherapy under the protocol.Contours from the clinical dataset were used for treatment planning and analysis and additional contours of heart substructures were completed by an oncologist.^47^ Table 1 lists a few patient characteristics.

**Table 1.** Patient and plan characteristics. Plan characteristics list are optimization type: Single field optimization (SFO), multifield optimization (MFO), and beam angles

Patient	Sex	Age (years)	CTV volume (cc)	1 Field Plan	2 Field Plan(s)	3 Field Plan
1	Female	15	241	SFO: 0°	SFO: 10° 350°	MFO: 10° 350°, 180°
2	Female	14	30	SFO: 0°	SFO: 10° 350°	SFO: 10° 350°, 180°
3	Male	17	194	SFO: 0°	SFO: 10° 350° SFO: 30° 330°	SFO: 10° 350°, 180°

### Treatment planning

We created three or four proton plans for each patient to investigate how the beam arrangement affected the LET and vRBE distributions (Eclipse v13.7, Varian Medical Systems, Palo Alto, CA). For all patients, plans with 1, 2, and three fields were created, and for one patient who was male, a ‘wide’ 2-field plan was also created (30 and 330 degrees) due to lack of breast tissue (Table 1).Beams were chosen with these limited angles to follow a clinically realistic configuration that avoids entrance dose through the lungs and breasts (for females).

**Figure 1.**
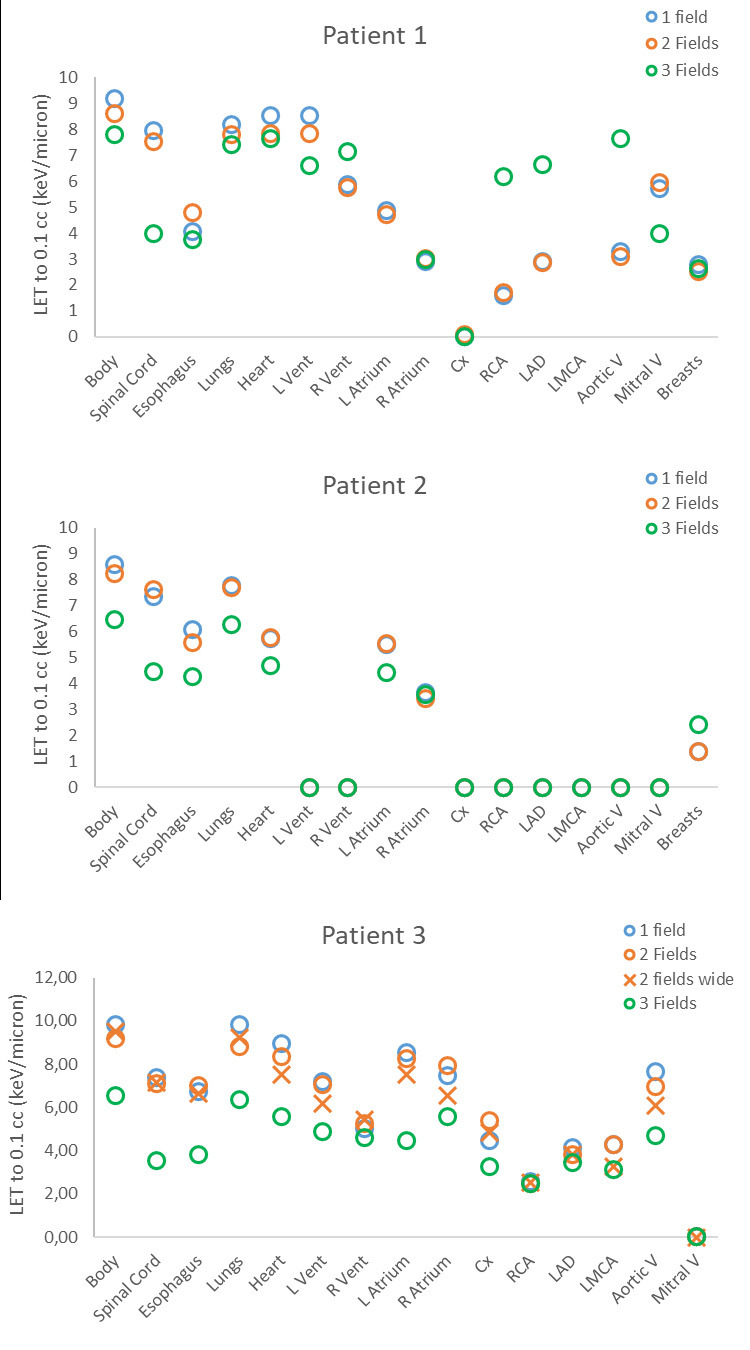
Near-maximum (0.1 cc) dose-averaged LET for OARs for different beam arrangements for the 3 patients in this study. OARs are listed in the order: body, spinal cord, esophagus, lungs, heart, left ventricle (L Vent), right ventricle (R Vent), left atrium (L Atrium), right atrium (R Atrium), circumflex artery (Cx), right coronary artery (RCA), left anterior descending artery (LAD), left main coronary artery (LMCA), breasts (for females). Data are missing for the LMCA for patient one is due to the size of the structure being too small.

Treatment planning was performed to a prescription dose of 19.8 Gy in 11 fractions using robust optimization to the CTV, assuming an uncertainty of 3.5% in CT calibration and 5 mm in positioning. For most plans, single field optimization (SFO) was used; however, for patient 1, the target surrounded the heart and multifield optimization (MFO) was used for the 3-field plan to avoid entrance dose through the heart by removing the region near the heart from the beam-specific planning volumes (Table 1, Figure 1). Priorities during optimization were first CTV coverage, and second to reduce the dose to the lungs and the heart as much as possible. Cardiac chambers and cardiac arteries were contoured for analysis but were not used during optimization. Robust evaluation was performed with the same uncertainty assumptions and plans were considered acceptably robust if 98% of the CTV received at least 95% of the prescription dose for 10/12 uncertainty scenarios. The dose grid for calculation in the treatment planning system (TPS) was 2.5 mm.

### LET and variable RBE calculation

Plans were exported, pre-processed for file compatibility, and imported to the in-house Monte Carlo (MC) system AUTOMC.^48^AUTOMC is based on Geant4 (v10.3.2)^49^ and Gate (Geant4 Application for Emission Tomography, v8.1) (RTIon v1.0)^50,51^ and commissioned for the ProBeam delivery system (Varian Medical Systems).The physics list was set to QGSP_BIC, voxels were 2 mm, and cuts were 0.1 mm for electrons and default settings forall other particles. Physical dose to material was scored and converted to physical dose to water,^52^ and dose-averaged LET to water for primary and secondary protons was scored using the ‘GetElectronicStoppingPowerDEDX’ method.^53^ The number of histories was scaled to achieve an approximate uncertainty level of 1% in dose in the high dose region.^54^

Post-processing of output files was completed in MATLAB (R2018b, MathWorks, Inc., Natick, MA). For plans with multiple beams, LET matrices from each beam were combined into one matrix using weighting factors of the relative dose to the voxel from each beam. LET matrices were thresholded to regions of at least 5% of the maximum dose to remove voxels containing very few particles and low clinical relevance. Then, matrices of physical dose and LET were used to obtain vRBE using the McNamara model^26^ with an assumed α/β of 2 Gy.^55^ For one plan, additional vRBE results were analyzed assuming additional α/βs of 3 Gy and 10 Gy for a sensitivity analysis. Finally, matrices of LET, physical dose, vRBE, and vRBE-weighted dose were converted to DICOM format and imported back into the TPS for visualization and analysis.

### Comparison metrics

To compare how these distributions changed for different beam arrangements with respect to the OARs, metrics were extracted for the body, lungs, spinal cord, esophagus, breasts (for females), heart, heart chambers (left and right atria and left and right ventricle), aortic valve, mitral valve, and coronary arteries (right coronary artery (RCA), left main coronary artery (LMCA), left circumflex (Cx), and left anterior descending (LAD)).

Specifically, for LET comparisons, near-maximum (D_0.1cc_) LET values were extracted. For dose comparisons, mean and near-maximum dose values were analyzed for the TPS dose with a fixed RBE of 1.1, MC dose with a fixed RBE of 1.1, and MC dose with vRBE.The values for near-maximum metrics were first extracted and then compared, so spatial information was not preserved. To perform analysis with spatial information preserved, we also created voxel-by-voxel dose difference maps, and we visually examined the overlap of regions of 80% physical dose from MC and regions of LET greater than or equal to six keV/μm.

## Results

All treatment plans had acceptable coverage and robustness (for the TPS dose) with the criterion of 98% of the CTV receiving at least 95% of the prescription dose for 12/12 uncertainty scenarios for all plans, except for the ‘wide’ 2-field plan for patient 3, which fulfilled the criteria for 10/12 uncertainty scenarios.

**Figure 2.**
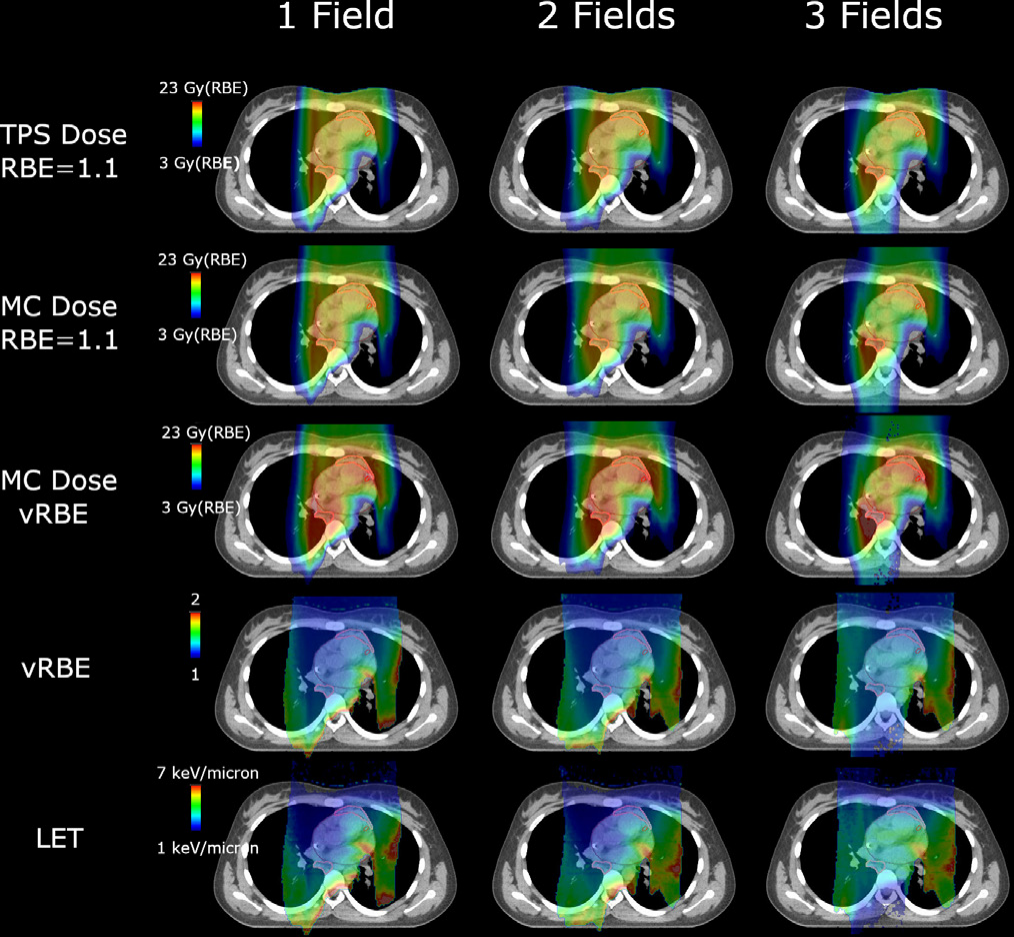
Distributions of TPS dose (RBE of 1.1), MC dose (RBE of 1.1), MC dose (with vRBE), vRBE, LET for patient 1 for 3 plans with 1, 2, and 3 fields, respectively. Abbreviations: Treatment planning system (TPS), Monte Carlo (MC), relative biological effectiveness (RBE), variable RBE (vRBE), and linear energy transfer (LET). Red regions in the vRBE and LET color wash distributions can be seen where the protons are at the end-of-range, but do not always correspond to regions of high physical dose.

We found that LET varied slightly with beam arrangement (Figure 1). In general, more beams decreased the near-maximum LET in OARs, but not always. Overlap of regions of 80% dose and high LET (≥6 keV/μm) were evaluated visually. For most plans there was no overlap. For two plans, a few voxels overlapped, but the regions were very small.Patient one had a region of overlap for the one-field plan of 0.004 cc in muscle, and patient three had a region of overlap for the one-field plan of 0.11 cc located in the vertebra, carina, and descending aorta (Figure 2). Details of mean and near-maximum LET for all patients can be found in the appendix (Supplementary Table 1).

Figure 2 shows an example of the TPS dose with a fixed RBE of 1.1, MC dose with a fixed RBE of 1.1,MC dose with vRBE, vRBE, and LET. LET and vRBE distributions increased at the end of range. It can also be seen that some of the regions of high LET and vRBE in the one- and two-field plans near the end of range were mitigated in the three-field plan that contains a posterior beam. Despite these differences, the vRBE-weighted dose distribution was very similar to the fixed RBE distributions for all plans due to the regions of high vRBE corresponding to regions of low dose.

**Figure 3.**
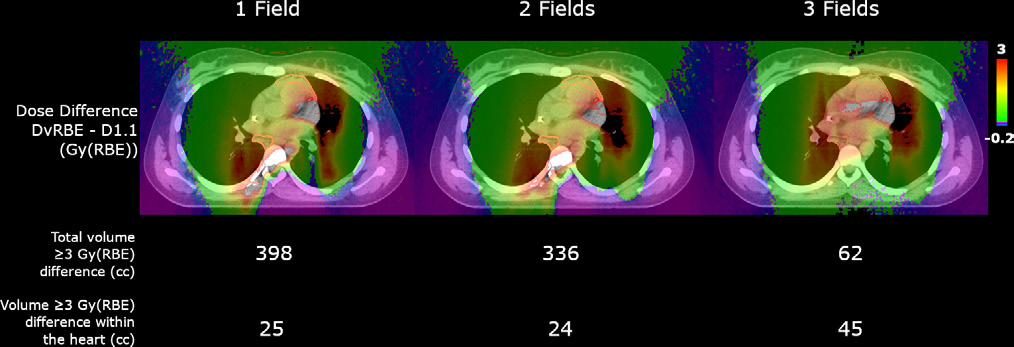
Dose difference distributions (*i.e.,* Δdose) and volumes with differences greater than or equal to 3 Gy (RBE) for patient one for plans with 1, 2, and 3 fields. Dose differences were calculated as a voxel-by-voxel subtraction of dose calculated with variable RBE (DvRBE) minus dose calculated with a fixed RBE of 1.1 (D1.1). The dose difference scale is from −0.2 to 3 Gy (RBE) (regions of black or clear are beyond the scale; see Figure 3 for higher scales) and the CTV is shown in pink and the left anterior descending artery (LAD) is shown in red.

Voxel-by-voxel dose-difference (*i.e.,*.Δdose) distributions and volumes with a dose-difference of greater than 3 Gy (RBE) are shown in Figure 3 for patient 1. While the total volume with a Δdose greater than or equal to 3 Gy (RBE) was reduced with three fields, the volume inside the heart was increased. Figure 3 in the supplementary material shows the one field plan with scales showing values above 3.5 and 4 Gy (RBE) to highlight the highest Δdose regions.

**Figure 4.**
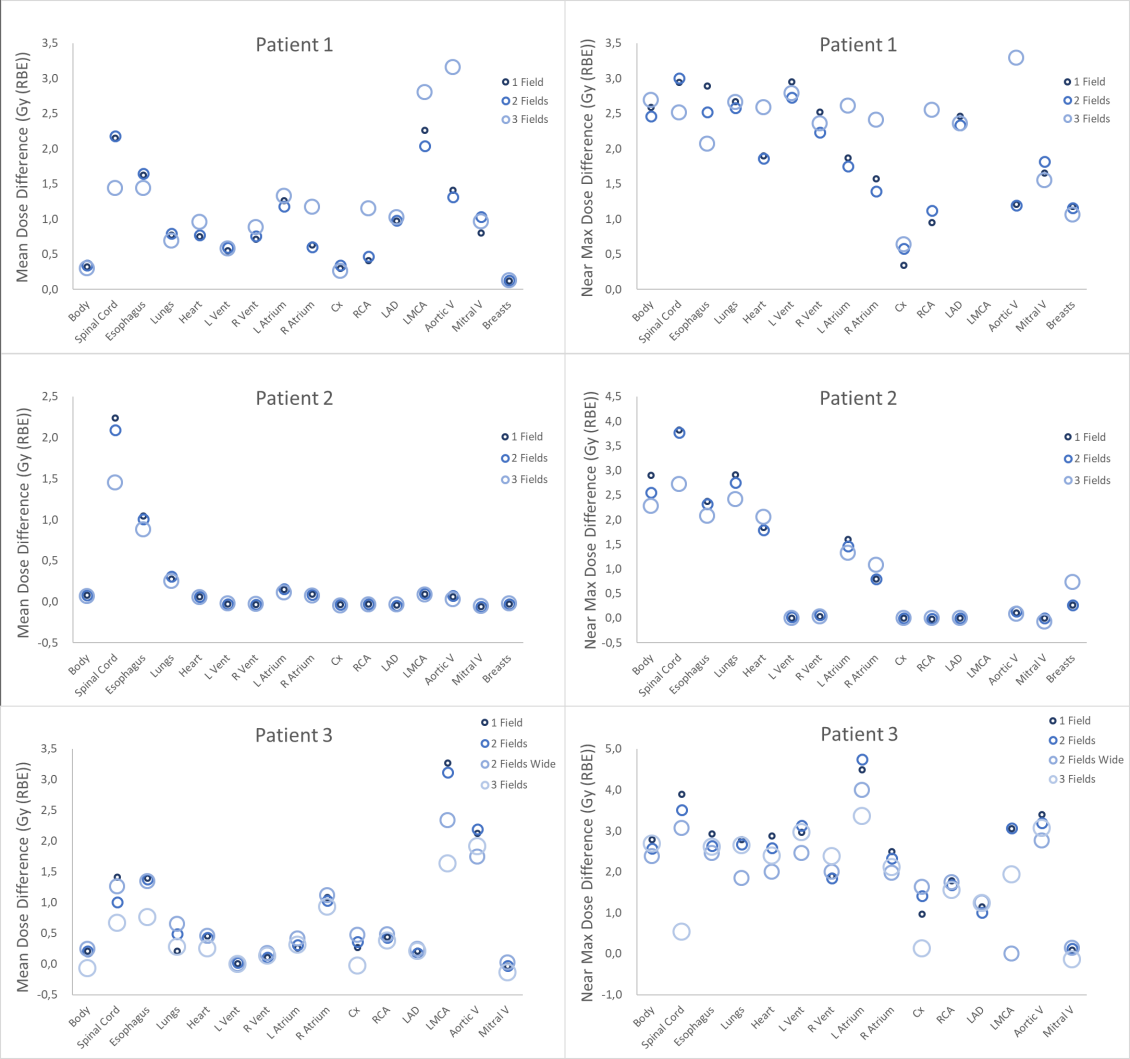
Mean and near-maximum (0.1 cc) dose differences (Δdose) between MC dose with either fixed RBE of 1.1 or vRBE and the TPS dose with fixed RBE of 1.1 for patient 1. OARs are listed in the order: body, spinal cord, esophagus, lungs, heart, left ventricle (L Vent), right ventricle (R Vent), left atrium (L Atrium), right atrium (R Atrium), circumflex artery (Cx), right coronary artery (RCA), left anterior descending artery (LAD), left main coronary artery (LMCA), breasts (for females).

Δdose in mean and near-maximum doses to OARs relative to the TPS dose with 1.1 are shown in Figure 4. Δdose values were generally larger for the near-maximum doses, with many values in the range of 2 to 3 Gy (RBE) and a maximum difference of 4.75 Gy (RBE) in the left atrium for patient 3 (Figure 4, bottom right panel). Increasing the number of fields sometimes reduced the differences, but not consistently. Further details of the dose metrics can be found in the appendix (Supplementary Material 1).

**Figure 5.**
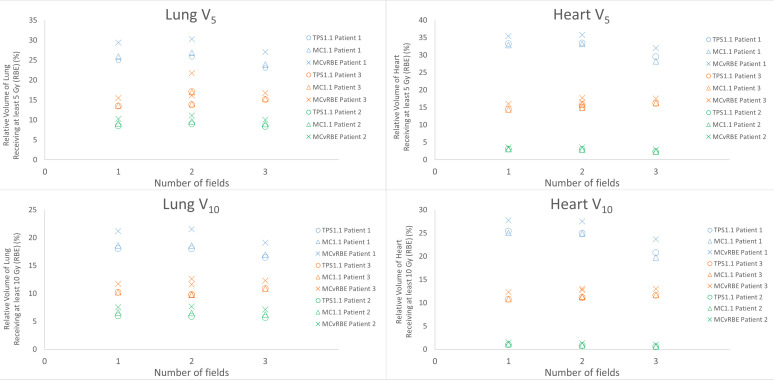
Dose-volume histogram (DVH) metrics for the lung and heart for all three patients by the number of fields. Doses were calculated using the TPS with a fixed RBE of 1.1 (TPS1.1), Monte Carlo with a fixed RBE of 1.1 (MC1.1), and Monte Carlo with a variable RBE (MCvRBE). For patient 3, both two-field plans are plotted.

DVH metrics for the relative volume of the lung and heart receiving at least 5 Gy (RBE) and 10 Gy (RBE) are shown in Figure 5. Dose metrics were improved for the three-field plan for patient 1, who had the largest doses to the lung and heart, but there was no clear association for the other two patients.

**Figure 6.**
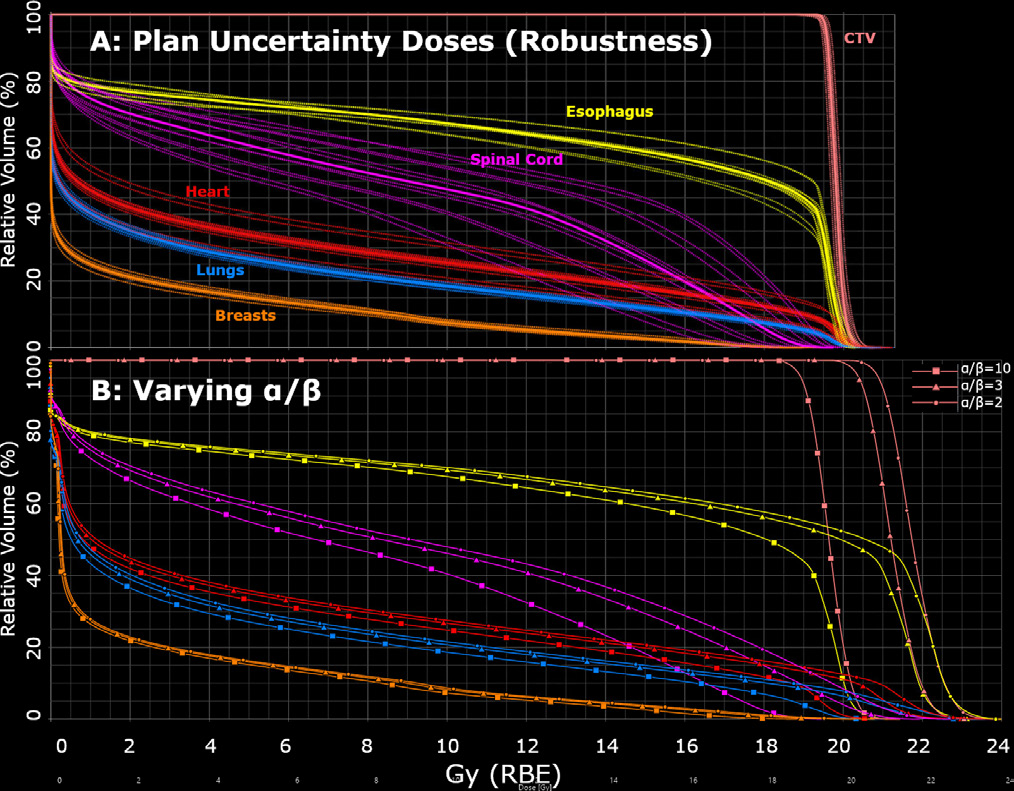
Dose-volume histograms (DVHs) for patient 1, 2-field plan, showing how differences in the DVH from robust analysis (plan uncertainty doses, calculated with the treatment planning system, assuming an RBE of 1.1) (**A**) compared to the differences from varying the α/β (2 Gy (default value for this study), 3 Gy and 10 Gy), which is used in the calculation of a variable RBE (Monte Carlo doses) (**B**).

To investigate the impact of uncertainties in these calculations, dose-volume histograms (DVHs) were calculated and compared for both uncertainties related to the Hounsfield unit (HU) and positioning (3.5% and 5 mm,i.e. plan uncertainty doses to evaluate robustness in the TPS) and uncertainties related to the choice of α/β in the calculation of variable RBE (2 Gy (default value for this study), 3 Gy, and 10 Gy) (Figure 6). The spread in the DVH curves was roughly similar, except for in the high dose region, where varying the α/β had a larger impact.

## Discussion and conclusion

In this study, we found that increasing the number of beams decreased the near-maximum LET in OARs for adolescent patients with mediastinal Hodgkin lymphoma; however, we did not find a consistent corresponding reduction in the Δdose to OARs with increasing number of beams. This could have been due to the choice of beam angles, which were limited to relatively anterior and posterior directions. Furthermore, the addition of the posterior beam increased the Δdose for the heart and some heart substructures for patient 1, who had the highest heart dose. This could be due to a larger volume of the heart being exposed to protons at the end of range because of the MFO strategy to avoid entrance dose to the heart. Some of the near-maximum doses to OARs in this study were approximately10–15% of the prescription dose higher for the variable RBE-weighted dose compared to the fixed 1.1 RBE-weighted dose.That could be potentially clinically significant, especially if near-maximum doses to OARs near the target are of particular importance for a specific plan evaluation or for considerationfor referral for proton therapy versus photon therapy (*e.g.,* dose to the LAD or heart values). Higher radiation doses to the heart or subregions of the heart correlate with morbidity and mortality,^6,56–59^ and this study highlights a need for further development of tools to be able to consider regions of high LET and its impact on variable RBE-weighted dose in clinical practice.

Tseng et al.^60^ compared photon therapy and proton therapy for patients with mediastinal lymphoma using both variable and fixed RBE. They used anterior or anterior oblique beams ( ± 30 degrees) for treatment planning but did not investigate the dependence of their results on the number or arrangement of the beams. They found median differences in maximum dose (Δdose) (vRBE minus fixed 1.1 RBE) of 1 Gy to the spinal cord and −0.2 Gy for the esophagus. They concluded that, for the patients in their study, proton therapy retained a dosimetric advantage above photon therapy, despite the effect of variable RBE. However, in contrast to our study, maximum (or near-maximum) doses to the heart or heart substructures with vRBE and fixed RBE were not compared.

Other studies have investigated LET and vRBE in proton therapy for other treatment sites. The magnitude of LET values in our study agree with one of the largest studies,^35^ which investigated different treatment sites, including thoracic patients. They found near-maximum values of LET in the OARs in the thorax of approximately 8–11 keV/μm on average, which agree well with our results of approximately 6–9 keV/μm. Ödén *et al* ^33^ found a relatively large difference in mean OAR doses (compared to using 1.1) for prostate cancer patients on the order of 2–3 Gy (RBE) and an increased probability of rectal toxicity. Another study from Carabe *et al* ^29^ investigated the impact of vRBE for prostate, brain, and liver patients, and found a large range of differences in the dose to 10% of the volume (D_10%_), ranging from differences of approximately 10 Gy (RBE) in OARs in the brain to differences as small as approximately 1 Gy (RBE) in the lung and healthy liver. The small differences between the results in these studies and in ours could be explained by the anatomical location of the target and the low prescription dose for Hodgkin lymphoma (which is also low for adult regimens, 20–30 Gy).

One limitation of this study is the uncertainty of the variable RBE model. There is a lack of data regarding validation of RBE models, which are based on *in vitro* data, and therefore little clinical consensus about choice of model. However, we chose a model that was built with experimental data from the relevant range,^27^ and other studies have shown that the McNamara model^26^ is a reasonably conservative choice with respect to OARs.^33,35^ We also explored the sensitivity of our results on the choice of α/β (Figure 5). Furthermore, this was a small study with only three adolescent patients and should not be considered exhaustive or conclusive, but rather exploratory.

In summary, we calculated LET and variable RBE-weighted dose for proton therapy for three adolescent patients with mediastinal lymphoma. While LET was reduced with increasing beam angles, we did not find a consistent reduction in the difference in variable RBE-weighted doses with increasing beam angles. Δdose (vRBE-weighted dose minus 1.1-weighted dose) varied by patient and organ but were on the order of about 0–1 Gy for mean doses and about 2–3 Gy for near-maximum doses (in the context of a prescription dose of 19.8 Gy). Therefore, until vRBE-weighted dose calculations are feasible in the clinical workflow, we recommend caution when near-maximum doses are near a constraint or could be a deciding factor in plan and/or treatment-modality selection. We cannot recommend any specific beam arrangement for the purpose of modulating the LET distribution; however, using multiple beams maybe be advantageous for other reasons such as plan robustness and quality.
